# Intraoral Surgical Approach and Evaluation of the Cellular Profile in a Granulomatous Reaction to a Foreign Body Induced by Calcium Hydroxyapatite in the Facial Region: A Case Report

**DOI:** 10.1111/jocd.70614

**Published:** 2025-12-26

**Authors:** Cíntia de Melo Braga, João Pedro Mapurunga da Frota Araújo, Gabriella Alves Julião Costa, Aira Letícia de Menezes Paiva, Paulo Goberlânio de Barros Silva, Luiz Andre Cavalcante Brizeno

**Affiliations:** ^1^ Departament of Dentistry Uninta Sobral Ceará Brazil; ^2^ Ceará Academy of Dentistry Fortaleza Ceará Brazil; ^3^ Department of Dentistry Unichristus Fortaleza Ceará Brazil

## 1 Introduction

Following the increase in demand for non‐invasive facial rejuvenation procedures, the demand for non‐surgical treatment options is also growing. Calcium hydroxyapatite‐based products function as a filler and biostimulator. They are biocompatible, biodegradable, and resorbable, containing calcium hydroxyapatite (CaHA) microspheres that provide volume replacement and stimulate endogenous collagen production [1].

CaHA was initially approved by the US Food and Drug Administration (FDA) in 2001 to be used as an injectable implant and to serve as a radiographic marker in soft tissue. Later, in 2006, the FDA approved Radiesse (Merz Aesthetics, Raleigh, NC) as a calcium hydroxyapatite‐based filler for use in nasolabial folds and in HIV patients associated with facial lipodystrophy resulting from chronic antiretroviral use [2].

Calcium hydroxyapatite in facial aesthetic procedures can be used in diluted form, essentially working as a collagen [3]. The most common and transient adverse effects of using CaHA are erythema, edema, pruritus, and hematomas. Among other complications, the formation of nodules has been observed, most often as a result of superficial placement of the filler. Other rare causes of nodule formation may include CaHA migration or foreign‐body granulomatous reaction (FBGR) formation [4].

FBGR consist of an inflammatory infiltrate composed of histiocytes and epithelioid cells. They can differ mainly by the proportion and arrangement of the inflammatory infiltrate, exudate, and presence of necrosis [5]. The treatment of nodules of CaHA is still a major therapeutic challenge. A number of treatment methods have been reported in the literature, depending on how long the lesion has evolved, including massage, rupture with a 22‐gauge needle, hyaluronidase, triamcinolone, and intralesional antimitotics, and, in some cases, follow‐up. However, these forms of treatment, depending on each case, may not be totally effective. Surgical excision is one of the options, but depending on the location of the lesion and the type of FBGR, it may result in unsightly scars or difficulty in completely removing the lesion [6].

Therefore, this report proposes a technique for removing FBGR caused by hyperdiluted caHA via the intraoral route and analyzing the profile and proportion of inflammatory cells in the lesion.

## 2 Methodology

### 2.1 Ethical Aspects

This is a case report carried out at the orofacial harmonization specialization clinic of the Ceará Academy of Dentistry in Fortaleza (Ceará). The study was approved by the Ethics and Research Committee of the Cearense Academy of Dentistry under opinion no. 6.897.595.

### 2.2 Histopathological Analysis

After the excisional biopsy, the specimen was fixed in 10% formalin for 24 h, dehydrated, diaphanized using xylene, and impregnated with paraffin. The specimen was cut into 4‐μm‐thick sections and stained with hematoxylin and eosin (HE). The sections were analyzed using an optical microscope (Motic BA310 microscope equipped). The analysis and histopathological report were carried out by a pathologist from the oral pathology laboratory at Christus University (Fortaleza—CE).

### 2.3 Analysis

The 3‐μm‐thick sections were placed on silanized slides and processed. The samples were deparaffinized, rehydrated, and subjected to antigen retrieval using ticitrate buffer (pH 6.0). To inactivate the endogenous peroxidase, the specimens were incubated (10 min; room temperature) with 6% H2O2 in methanol (1:1), washed with Tris buffer at pH 7.6, incubated for 1 h (room temperature) with primary antibodies (Ab) directed against CD 68 (Dako Systems), CD 20 (Dako Systems), CD8 (Dako Systems), and CD3 (Dako Systems), washed, and then incubated (30 min; room temperature) with biotinylated immunoglobulin (Ig; DAKO E0468) and streptavidin (DAKO P0397). Subsequently, the chromogen diaminobenzidine (DAKO K3469) was applied to the specimens for 10 min.

Mayer's hematoxylin was used as a counterstain, then the specimens were dehydrated (using ethanol and xylene) and covered with coverslips using a permanent mounting medium. As a negative control, the primary antibody was omitted to assess the presence of any possible interference reaction between the tissue cells and the streptavidin–biotin–peroxidase complex that could compromise the results.

**FIGURE 1:**
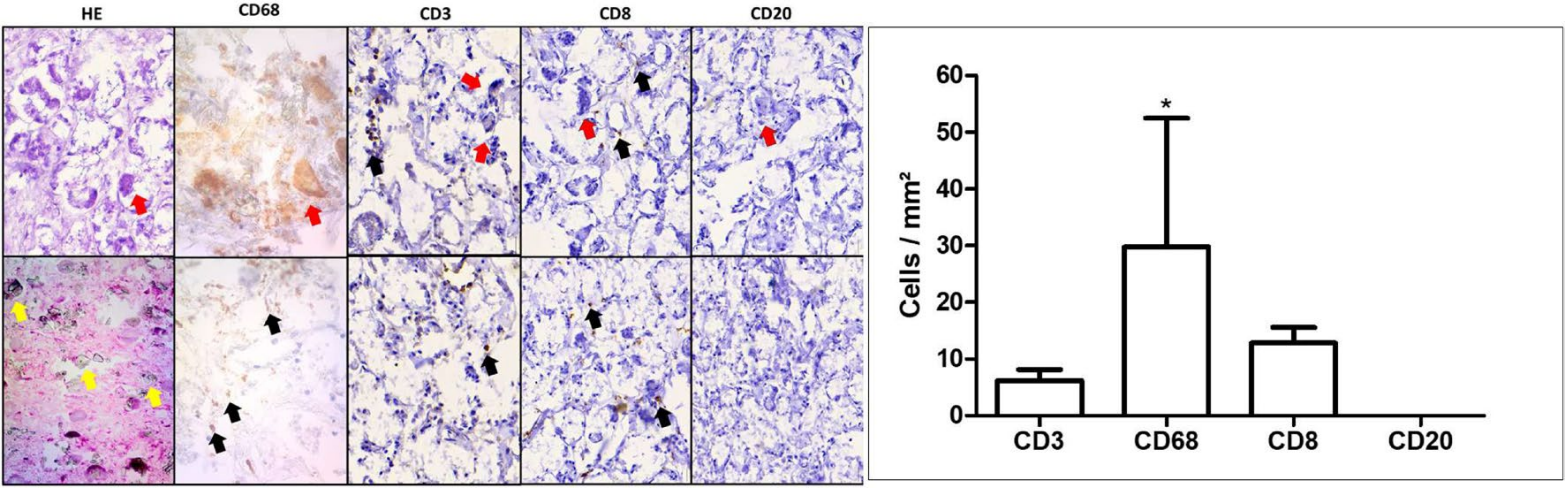
Histological and inflammatory cell profile of excisional biopsy of RGCE. Magnification = 400×; HE = Hematoxylin–eosin; H‐DAB = hematoxylin‐diaminobenzidine (CD68, CD3, CD8, and CD20). Yellow arrows = material; black arrows = mononuclear cells showing immunoexpression for the marker studied; red arrows = multinucleated giant cells.

Quantitative immunohistochemical evaluation was carried out using five randomly selected photographed fields in regions with the highest concentration of immunostained cells located in the connective tissue (using a Motic BA310 microscope equipped with a Moticam 2000 2.0 M Pixel camera and Motic 2.0 software; 400× magnification). Cells with cytoplasmic or nuclear immunolabeling were counted using the “Cell Counter” plugin tool in ImageJ (RSB) software (Figure 1) [7].

### 2.4 Statistical Analysis

For a multiple comparison of the immunohistochemical results, the mean and standard deviation of the five selected fields were calculated, and the repeated‐measures ANOVA test was used. In all statistical analyses, the critical level for rejecting the null hypothesis was considered to be less than 5% (*p* < 0.05). Statistical analyses were carried out using the GraphPad Prism 5.0 statistical program.

## 3 Case Report

DAM, a 38‐year‐old female patient, attended the Orofacial Harmonization Improvement Clinic at the Ceará Academy of Dentistry (ACO) on 29/07/2020 complaining of dynamic wrinkles in the upper third of the face and sagging in the middle third. A treatment plan was established with the application of botulinum toxin in the upper third, one session of PRF (fibrin‐rich plasma), and biostimulator. During the anamnesis, the patient did not report any underlying disease, nor any medication use.

Clinical examination revealed the presence of dynamic wrinkles in the upper third of the face and moderate sagging in the middle third of the face and cervical region; 18 units of botulinum toxin were applied to the upper third of the face (29/07/2020). On 30/08/2020, the botulinum toxin was reviewed (without complications), and a subdermal application of PRF was carried out on the middle third of the face and microneedling with PRF on all facial thirds. On 16/12/2020, the patient was re‐evaluated and said she was satisfied with the procedures carried out; however, the complaint of sagging remained, so calcium hydroxyapatite (Rennova Diamond) hyperdiluted at 7.5% (1,25 mL of 30% hydroxyapatite +2.75 mL of saline +1 mL of 2% lidocaine without vasoconstrictor), totaling a final volume of 5 mL of product, was applied to the middle third of the face (2.5 mL on each side) subcutaneously using a 22G cannula.

After the procedure, the area was massaged to accommodate the material and the patient was instructed to perform the massages at home for 5 days, for 5 min, 5 times a day. On February 20, 2021, the patient contacted the professional (a postgraduate HOF (Facial Harmonization) student) who carried out the procedure, reporting the presence of a palpable nodule on the left jugal mucosa, without pain, erythema, or any other symptoms, and the patient was scheduled for an evaluation.

On visual inspection of the extraoral region mentioned by the patient, nothing noteworthy or aesthetically inconvenient was observed. On palpation, a hardened, sessile nodule measuring approximately 1.5 cm was painless.

**FIGURE 2:**
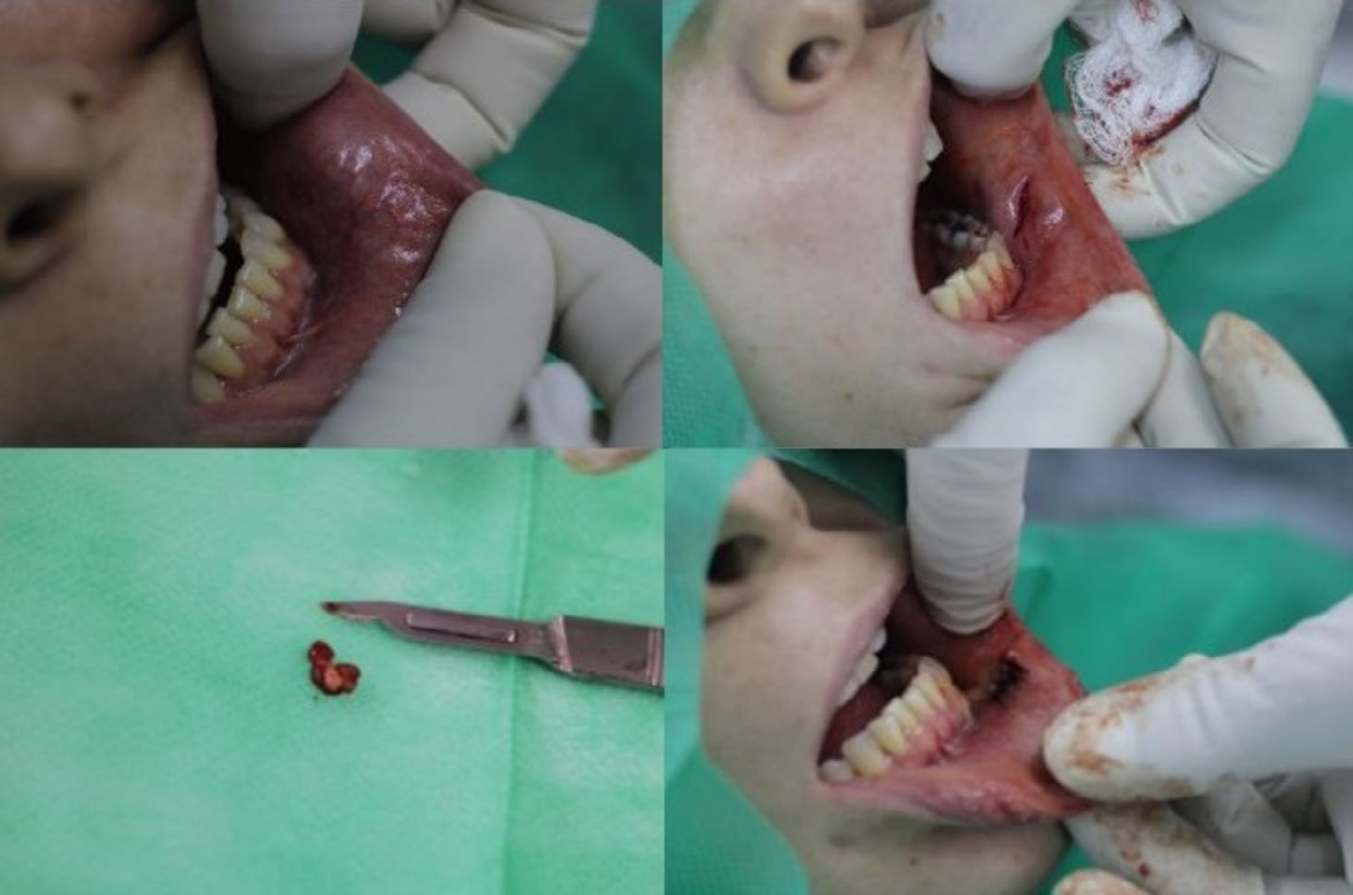
Location of the lesion on the jugal mucosa and surgical bed after removal of the lesion.

Intraoral examination showed a whitish nodule when pressing on the area; the lesion had no infectious or acute inflammatory aspect. The clinical diagnosis was foreign body granulomatous reaction due to CaHA. In the same session, 100 UTR (Units of hyaluronidase) of hyaluronidase was applied (20 UTR intralesionally and 80 UTR around the nodule, using a 30G × 13 mm needle and a 1 mL syringe). A dilution of 2000 UTR of hyaluronidase +4 mL of sterile physiological solution +1 mL of 2% lidocaine without a constrictor vessel was used. The area was massaged and a new assessment scheduled. After reassessing the patient, it was observed that the lesion had not decreased in size or appearance. As the lesion was palpable in the intraoral region (Figure 2), it was decided to remove it surgically using this access route. On April 27, 2020, an excisional biopsy of the lesion was performed, and the specimen was sent for anatomopathological examination.

For surgery, the region was anesthetized using 1.5 tubes of 2% lidocaine with 1:100.00 epinephrine, a linear incision was made, the adjacent tissues were divided with straight scissors, and once the lesion had been located, it was seized with Allis forceps and removed, followed by suturing of the surgical site (Figure 2).

Dipyrone 1 g was prescribed for 3 days to control the pain, and the suture was removed after a week. The patient is being followed up without any complications and with definitive resolution of the lesion.

Immunohistochemistry was also carried out for CD68 (macrophage), CD3 (TCD3 lymphocyte), CD20 (B‐lymphocyte), and CD8 (cytotoxic T‐lymphocyte), and the respective immunostained cells were counted to assess the cellular profile of the lesion (Figure 1).

## 4 Discussion

Minimally invasive facial rejuvenation treatments are gaining popularity due to their lower risk and shorter postoperative recovery time compared to traditional surgical procedures. Biostimulating agents are one of these modalities, being used both as alternatives and as complements to other non‐surgical techniques. The possibility of FBRG is one of the adverse effects that can occur with the use of these products. In the present report, the treatment proposed for FBRG was surgical with intraoral access, and the cellular profile of the lesion showed a greater number of macrophages when compared to T‐lymphocytes.

The application of calcium hydroxyapatite can cause short‐term transient adverse effects, such as erythema, edema, hematoma, and pain, which were not reported in the immediate postoperative period. However, some adverse effects can manifest later, such as FBRG. In this study, the lesion was reported 2 months after treatment [8].

The incidence of this type of reaction is considered rare, depending on the type of material to be injected, its chemical nature, commercial presentation, and degree of purity. Manipulated products or those not certified by regulatory agencies in relation to strict quality control processes can generally present a higher risk of complications [9]. In this study, the biostimulator used had 30% CaHA dispersed in Carboxymethylcellulose Gel and is duly certified and approved for injectable use by the National Health Surveillance Agency (Anvisa).

Nodulation and migration of calcium hydroxyapatite to the perioral region have already been observed. Previous studies have reported similar foreign body granulomas in 8 cases on the lips or buccal mucosa [10]. In the present case, there may have been displacement from the “chambers” region to the jugal mucosa.

The “migration” of dermal fillers can also be related to the injection of a large volume and high pressure, or gravity. Such mechanisms can result in the occurrence of nodules in deeper layers than the original injection, or in the jugal mucosa, when the application occurs in the overlying skin [11]. In this case, muscle movement or the deepening of the cannula may have contributed to the agglutination and migration of the particles, as the volume used complied with the recommendations for applying the material (retroinjections of 0.1 mL in the temples, 0.2 mL in the middle third of the face, and 0.1 mL in the lower third, an average spacing of 1 cm between them) using a 1‐mL syringe and 22G cannula (Figure 1).

Nodules caused by calcium hydroxyapatite occur in 7.0%–12.4% of cases, with early or immediate resolution with massage and late resolution through intralesional corticosteroid injection [10]. In some cases of unresponsive granuloma to conservative therapies, surgical removal is used as in the present study.

Surgical excision using a direct approach to the focus of the lesion has provided resolution in some types of FBRG; however, it can sometimes leave scars in aesthetic areas [12]. In general, it is not usually the first treatment option for some FBRGs due to the difficulty of completely removing the lesion. FBRGs with little capsule and tissue formation after collagen injection, HA (Hyaluronic Acid), or compact CaHA nodules may not respond to intralesional corticosteroids and antimitotics, and surgical removal is probably the method of choice for these cases [7]. This fact justified our surgical approach in the present study, as it was a localized, compact lesion that allowed intraoral access; therefore, without the risk of residual scarring in the patient's facial region, and prior to the surgical procedure, a more conservative treatment option was attempted, but without therapeutic success.

After excision, the specimen should be sent for histopathological analysis because intraoral lesions can raise suspicions of other pathologies such as mucoceles, malignant and benign salivary gland tumors, or soft tissue [13].

Histopathological examination showed the deposition of crystalloid and refringent material, compatible with a foreign body, the presence of multinucleated giant cells, and a mononuclear inflammatory infiltrate, labeled as a reaction to a foreign body (crystalloid material) (Figure 8).

This cellular behavior reported in our study is typical of a GBER (Glabella, Nasolabial folds, Malar region, and Jawline). The literature has pointed to the possibility of GER (Granulomatous Foreign Body Reaction) caused by CaHA showing differences in the profile of inflammatory cells when compared to other materials, including those caused by HA [7, 14].

CaHa FBGR would generate a more macrophagic response, while those caused by HA would generate a more lymphocytic infiltrate, [15] a fact observed in the present report where we observed a greater number of macrophages when compared to TCD3 and TCD8 lymphocytes. The hypothesis for this difference may be related to the question of how immunologically inert the injected material is, and this can be influenced by various reasons, such as the composition and quantity of the material, the shape and size of the particles, their biodegradability, and in the case of HA, the concentration, degree, and cross‐linking technology [7, 15].

No B‐lymphocytes were marked in the lesion, which is also to be expected as the development of foreign body granulomas most often represents a type IV hypersensitivity reaction to an immunologically inert material. The aim of the reaction is to encapsulate and isolate material that cannot be removed immediately by enzymatic degradation or phagocytosis. It is defined by the presence of T‐lymphocytes and histiocytes (macrophages), which respond to various chemical mediators of cell damage [16].

The exact reason why FBRG caused by CaHA often does not respond satisfactorily to treatment with oral or intralesional corticosteroids is not yet known in the literature [7]. Could this distinct cellular pattern be related to the therapeutic response? One hypothesis is that corticosteroids prevent proliferation, clonal expansion, and induce apoptosis of T‐lymphocytes [17]. This may contribute to the better response of granulomas caused by HA to treatment with these drugs, as they have a more lymphocytic cellular microenvironment [7, 15], as well as differences in the pattern of cytokines released in the lesional microenvironment [17]. Therefore, understanding the pathophysiology of FBRG caused by different materials is crucial for establishing more assertive therapies, although further studies are needed to clarify the hypotheses raised.

## 5 Conclusion

The case reported presented a definitive and effective treatment for FBRG caused by CaHA, with the advantage of not leaving a scar in an aesthetic area of the face. The inflammatory cell profile of the lesion had a presence of TCD3 and TCD8 lymphocytes, an absence of B‐lymphocytes, and a greater number of macrophages. The intraoral access technique and the cellular profile described may represent a good alternative to avoid scarring in aesthetic areas and to better understand the pathophysiology of granulomas caused by CaHA and establish better therapeutic approaches; however, more studies are needed to confirm the hypotheses. The patient remains under observation with no signs of residual lesions.

## Author Contributions

Cíntia de Melo Braga and João Pedro Mapurunga da Frota Araújo did the clinical management of the patient, data curation, writing – review and editing. G.A.J.C. and Aira Letícia de Menezes Paiva: conceptualization, data collection, writing – original draft preparation. They read and approved the final version of the article. Paulo Goberlânio de Barros Silva and Luiz Andre Cavalcante Brizeno: supervision and final manuscript approval.

## Funding

The authors have nothing to report.

## Ethics Statement

The study was approved by the Ethics and Research Committee of the Cearense Academy of Dentistry under opinion no. 6.897.595.

## Consent

Written informed consent was obtained from the patient to publish this case report in accordance with the journal's patient consent policy.

## Conflicts of Interest

The authors declare no conflicts of interest.

## Data Availability

Research data are not shared.
